# Six years of ecosystem-atmosphere greenhouse gas fluxes measured in a sub-boreal forest

**DOI:** 10.1038/s41597-019-0119-1

**Published:** 2019-07-05

**Authors:** Andrew D. Richardson, David Y. Hollinger, Julie K. Shoemaker, Holly Hughes, Kathleen Savage, Eric A. Davidson

**Affiliations:** 1 Center for Ecosystem Science and Society, Northern Arizona University, Flagstaff, AZ 86011 USA; 2 School of Informatics, Computing, and Cyber Systems, Northern Arizona University, Flagstaff, AZ 86011 USA; 3 USDA Forest Service, Northern Research Station, Durham, NH 03824 USA; 4 Lesley University, Cambridge, MA 02138 USA; 5 University of Maine, Orono, ME 04469 USA; 6 Woods Hole Research Center, Falmouth, MA 02540 USA; 7 Appalachian Laboratory, University of Maryland Center for Environmental Science, Frostburg, MD 21532 USA

**Keywords:** Carbon cycle, Ecosystem ecology, Climate-change ecology

## Abstract

Carbon dioxide (CO_2_), methane (CH_4_), and nitrous oxide (N_2_O) are the greenhouse gases largely responsible for anthropogenic climate change. Natural plant and microbial metabolic processes play a major role in the global atmospheric budget of each. We have been studying ecosystem-atmosphere trace gas exchange at a sub-boreal forest in the northeastern United States for over two decades. Historically our emphasis was on turbulent fluxes of CO_2_ and water vapor. In 2012 we embarked on an expanded campaign to also measure CH_4_ and N_2_O. Here we present continuous tower-based measurements of the ecosystem-atmosphere exchange of CO_2_ and CH_4_, recorded over the period 2012–2018 and reported at a 30-minute time step. Additionally, we describe a five-year (2012–2016) dataset of chamber-based measurements of soil fluxes of CO_2_, CH_4_, and N_2_O (2013–2016 only), conducted each year from May to November. These data can be used for process studies, for biogeochemical and land surface model validation and benchmarking, and for regional-to-global upscaling and budgeting analyses.

## Abstract (summary)

**Table:** 

Design Type(s)	observational design • data collection and processing objective
Measurement Type(s)	greenhouse gas
Technology Type(s)	environmental monitoring
Factor Type(s)	greenhouse gas • temporal_interval
Sample Characteristic(s)	Howland Forest • forest ecosystem

Machine-accessible metadata file describing the reported data (ISA-Tab format)

## Background & Summary

Increases in atmospheric concentrations of carbon dioxide (CO_2_), methane (CH_4_), and nitrous oxide (N_2_O) are driving the radiative forcing of climate that has occurred since 1800^1^. While these increases are predominantly the result of human activities, significant exchanges of these gases occur naturally between terrestrial ecosystems and the atmosphere. For example, global photosynthetic uptake by terrestrial ecosystems (≈123 ± 8 Pg C y-1 as CO_2_, ref.^2^) is a massive flux, but at annual time scales under current climate conditions this uptake is largely offset by a comparable efflux of respiratory carbon back to the atmosphere. By comparison, anthropogenic emissions of carbon to the atmosphere (9.5 ± 0.5 Pg C y-1 as CO_2_, ref.^3^) are not offset by existing sinks. The increase in atmospheric CH_4_ during the industrial era—from 823 ppb in 1841^4^ to over 1800 ppb at present^5,6^—is attributed to both fossil fuel emissions and microbial emissions^5^. Importantly, soils can be either a CH_4_ sink or source. Anaerobic CH_4_-emitting microbes (methanogenic archaea) are commonly found in wetland environments, while aerobic CH_4_-consuming microbes (methanotrophic bacteria) are often found in upland soils. Soil processes are the dominant source of N_2_O, with fluxes from natural systems accounting for about 35% of global emissions^7^. N_2_O can be produced by microbes under both anaerobic (via denitrification) and aerobic (via nitrification) conditions^8^, although the bulk of N_2_O production occurs in waterlogged soils^9^. Agricultural practices (accounting for 25% of global emissions), fossil fuel combustion, and industrial activities further contribute to N_2_O emissions^7^. Reports of N_2_O consumption by soil microbes have been controversial^10,11^. Thus for each of CO_2_, CH_4_, and N_2_O, natural biological processes play an important role in the global budget.

Land-atmosphere fluxes and atmospheric concentrations of CH_4_ and N_2_O are orders of magnitude smaller than those of CO_2_. The atmospheric lifetimes of CH_4_ (12 y) and N_2_O (114 y) are also shorter than that of CO_2_ (5–200 y, see ref.^1^). But, as greenhouse gases, CH_4_ and N_2_O are particularly important because of their much higher radiative forcing effect^1^. This motivates efforts to better understand the spatial and temporal patterns of land-atmosphere CH_4_ and N_2_O flux, and the biotic and abiotic factors controlling these patterns.

Here, we describe a six-year data set characterizing greenhouse gas fluxes at Howland Forest, Maine^12^. Vegetation at Howland, which is located within the boreal-northern hardwood transition zone, is dominated by the conifers red spruce and eastern hemlock^13^. The climate is cold and continental, although summers are warm. Soils are generally Spodosols with high organic matter content^14^.

Tower-based measurements consist of ecosystem-atmosphere turbulent fluxes of CO_2_, CH_4_, H_2_O (latent heat), and sensible heat, made using the eddy covariance method and reported at 30-minute temporal resolution. While long-term CO_2_ and H_2_O flux measurements are now being conducted at hundreds of sites around the world^15^ (some of these records—including data from Howland^13^—extend 20 years or more), long-term tower-based measurements of CH_4_ fluxes have been made at comparatively few sites, and generally in the last decade. Beyond our tower-based CH_4_ measurements at Howland^16^, only a handful of other studies have been published for temperate^17–19^ and tropical^20,21^ forests. Much more attention has been paid to wetland systems^22–26^, which are generally strong sources of CH_4_. Previous analysis of our data has indicated that at an annual time step, Howland Forest switches from a weak CH_4_ source to a weak CH_4_ sink depending on hydrologic conditions during late summer^16^.

We have also conducted measurements of soil-atmosphere greenhouse gas fluxes using automated chamber-based methods^27,28^. Here we describe a complementary five-year (2012–2016) dataset of chamber-based measurements of soil CO_2_, CH_4_, and N_2_O (2013–2016 only) fluxes, conducted along a gradient of upland, transition (not measured in 2013), and wetland sites close to the main research tower. In 2015–2016, soil fluxes of all three gases were measured in sites representing all three soil drainage classes.

A subset of the dataset^29^ described here is available through the AmeriFlux data portal^30^. We have two goals in describing and distributing a more complete dataset via Figshare. First, we aim to document tower and chamber flux measurements (e.g., instruments, processing, and QC) that had not yet been fully described in our previous papers^12,16^. Second, we are making data publicly available that cannot otherwise be handled or distributed through the current AmeriFlux data distribution system. This includes a variety of important variables output through the flux processing software (variances and covariances, flux uncertainties, spectral correction factors, and trace gas time lags) as well as all of the chamber data.

These data will be of use for investigations into the factors controlling greenhouse gas fluxes; for validation of ecosystem, biogeochemical, and earth system models; and for upscaling and budgeting analyses. While the CH_4_ and N_2_O fluxes from Howland are small compared to other systems, we argue that to accurately estimate global budgets, it is as important to know where the fluxes are small as it is to know where they are large.

## Methods

### Study site

**Fig. 1:**
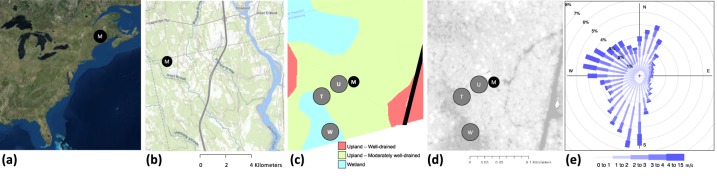
Location of the Howland AmeriFlux site. M denotes the Main Tower (US-Ho1). (**a**) Locator map, showing eastern North America; (**b**) Locator map, showing the ≈10 km surrounding the tower; (**c**) Locator map, with soil drainage classes, showing the ≈250 m surrounding the tower and the location of chambers in upland (U), wetland (W) and transition (T) topographic locations; (**d**) Locator map, with LiDAR canopy height measurements (light = high, dark = low) (horizontal scale is the same in panels (c,d); (**e**) Wind rose indicating the frequency distribution of wind speed and direction (in all directions, the median flux footprint peak occurs at a distance of ≈100 m from the tower).

**Fig. 2:**
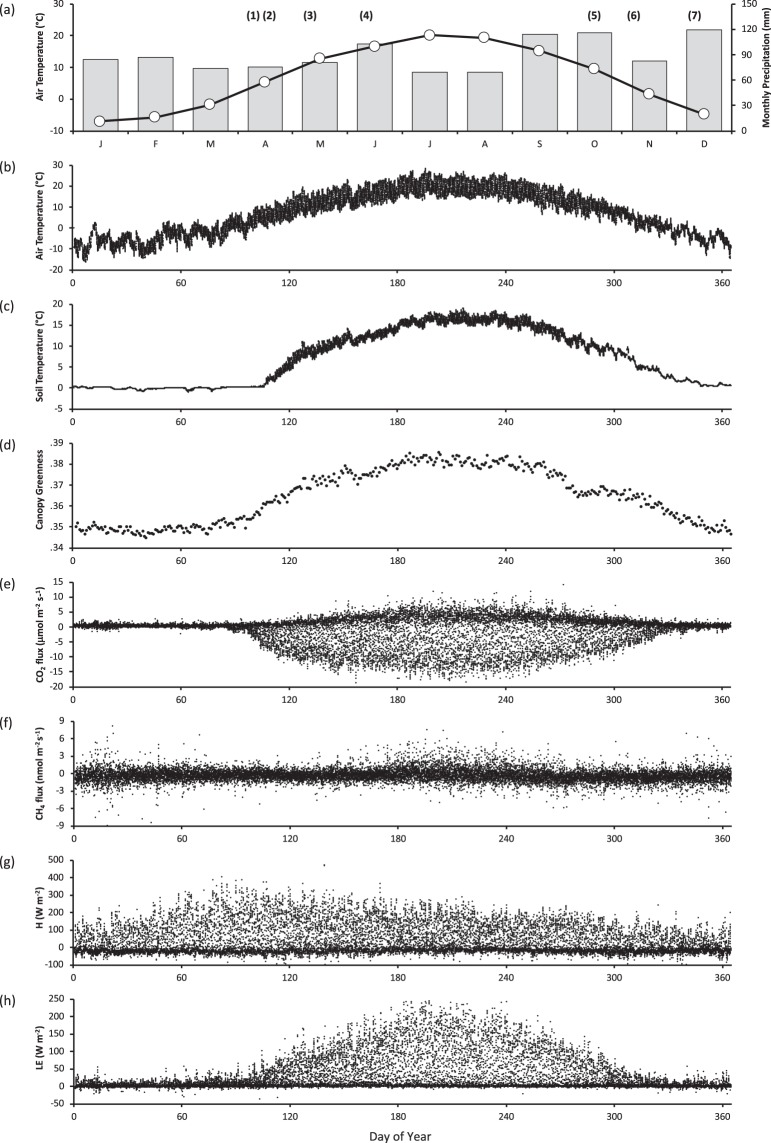
Seasonality of environmental variables and ecosystem fluxes at the Howland AmeriFlux site. Means calculated over the period 2012–2018. (**a**) Monthly air temperature (line) and precipitation (bars), in relation to key phenological events: (1) snow melt, (2) last frost, (3) budburst of deciduous trees, (4) budburst of evergreen trees, (5) deciduous trees drop leaves, (6) first frost, (7) first persistent snow; (**b**) half-hourly air temperature; (**c**) half-hourly soil temperature; (**d**) daily canopy greenness, derived from PhenoCam imagery; (**e**) half-hourly net ecosystem exchange of CO_2_; (**f**) half-hourly net ecosystem exchange of CH_4_; (**g**) half-hourly sensible heat flux (H); (**h**) half-hourly latent heat flux (LE).

Research was conducted at the Howland Forest AmeriFlux site located (Fig. 1) about 35 miles north of Bangor, Maine, USA (45.2041°N 68.7402°W, elevation 60 m above sea level) on forestland owned by the Northeast Wilderness Trust. The site sits at the southern ecotone of the North American boreal spruce-fir zone. Red spruce (*Picea rubens* Sarg.) and eastern hemlock (*Tsuga canadensis* (L.) Carr.) together account for about 70% of basal area, with other conifers (northern white cedar, Thuja occidentalis; balsam fir, *Abies balsamea*; and white pine, *Pinus strobus*) together accounting for 20% of basal area. Hardwoods, including red maple (*Acer rubrum* L.) and paper birch (*Betula papyrifera* Marsh.), together account for 10% of basal area^13^. Seasonality in leaf area index of the evergreen canopy is minimal; peak LAI during the growing season is about 5 m^2^ m^−2^. The undisturbed stand (mean age ≈120 y, maximum age ≈225 y; basal area 48 ± 17 m^2^ ha^−1^; canopy height ≈20 m) surrounding the “main tower” (one of four instrumented research towers at the site) is atypical of the regional landscape, where intensive forestry activities have taken place for over a century. Topography is flat to gently rolling. Soils range from well drained to poorly drained. Mean annual temperature is 6.1 °C and mean annual precipitation is 990 mm. The seasonal patterns of variation in environmental factors, phenology, and ecosystem-atmosphere fluxes are illustrated in Fig. 2. Climate, soils, and vegetation at the site are described in greater detail in earlier publications^12–14^ and documented in the AmeriFlux BADM (Biological, Ancillary, Disturbance and Metadata) file for this site (see “Additional Files” in the Data Records section, below). More recent publications comprehensively document the forest stand composition, structure, and growth^31,32^ in the vicinity of the main tower.

### Tower-based flux measurements

Continuous measurements of surface-atmosphere exchanges of CO_2_, H_2_O, and energy, made using the eddy covariance approach^33,34^, were initiated at the main Howland tower in 1995 and have been previously reported and fully documented^12,13^. Beginning in 2011, we expanded our measurement capabilities to include eddy covariance measurements of CH_4_ fluxes. An improved gas analyser for CH_4_ fluxes was installed in 2012^16^. Here we describe measurements from that instrument over the period 2012 to 2018.

Fluxes were measured at a height of 31 m with an instrument system consisting of a model SAT-211/3 K 3-axis sonic anemometer (Applied Technologies Inc., Longmont, CO, USA) and a fast-response CH_4_/CO_2_/H_2_O cavity ring-down spectrometer (model G2311-f, Picarro Inc., Santa Clara, CA, USA). Sampled air was pulled from the top of the tower through ~46 m of 4.8 mm (inner diameter) LLDPE (U.S. Plastics Corp., Lima, OH, USA) tubing (replaced annually), sheathed in flexible PVC pipe to minimize temperature fluctuations, using a vacuum pump (model MD4-NT, Vacuubrand GmbH, Wertheim, Germany) to maintain low cavity pressure and a flow rate ≥7 standard litres per minute. The distance between the air inlet and the sonic anemometer was less than 30 cm. All data, including concentrations of CH_4_ and CO_2_ reported as dry air mole fractions (mixing ratios), were recorded at 5 Hz on a data logger (model CR1000, Campbell Scientific Inc., Logan, UT, USA).

Raw, high-frequency data (available on request from D.Y.H.) were converted to 30 minute fluxes using the open source EddyPro® Eddy Covariance Processing Software, version 6.2.2 (LI-COR Biosciences, Lincoln, NE, USA)^35^. Turbulent fluxes calculated include sensible (H) and latent (LE) heat fluxes, as well as fluxes of CO_2_ (CO_2__flux) and CH_4_ (CH4_flux). The custom EddyPro settings used are summarized in Table 1. We processed the data in two batches: June 2012-June 2015 and June 2015-June 2018. The resulting files were concatenated in chronological order. The full EddyPro output file, with no filtering, is included here.

**Table 1:** Custom EddyPro settings used in processing Howland Forest data.

EddyPro option	Setting
Axis rotations for tilt correction	Double rotation
Turbulent fluctuation detrending	Running mean, 600 s time constant
Time lags compensation	Covariance maximization with default
Compensate density fluctuations (WPL)	No (trace gas concentrations recorded as dry air mole fractions)
Analytic correction of high-pass filtering effects	Yes, following Moncrieff^60^
Correction of low-pass filtering effects	Yes, following Ibrom *et al*.^61^
Correction for instrument separation	Yes, following Horst and Lenschow^62^
Flux footprint estimation	Yes, using the Kljun *et al*. model^63^
Random uncertainty quantification	Yes, following Finkelstein and Sims^45^

Unless specified below, default settings were used for all other options.

### Filtered tower-based flux measurements

We used the EddyPro output file to generate a filtered data set, also included here, which follows AmeriFux standard formats and which is recommended for most applications.

Following methods we have used at Howland for over 20 years^12,13^, we created a 14-bit QC flag that assessed each half-hour against a range of criteria we have found useful (Table 2). These include thresholds for windspeed, sonic anemometer temperature “spikes”, and sensor variance (insufficient variance likely indicating no turbulence or failed pump, excess variance indicating material on sonic transducers, system leaks or analyser malfunction). If a condition was true, the appropriate bit of the Howland QC flag was set to 1.

**Table 2:** Interpretation of custom quality flag descriptor and associated criteria.

Bit number	Meaning/interpretation	Frequency
1	*u* = −9999 (missing)	6.3%
2	Mean *u* < 0.5 m s^−1^	2.9%
3	Sonic anemometer *w* variance < 0.005	4.0%
4	Sonic anemometer *w* variance > 1.5	3.7%
5	Sonic anemometer *T* variance < 0.002	5.1%
6	Sonic anemometer *T* variance > 2.5	1.7%
7	Sonic anemometer *T* spikes > 150	1.2%
8	Picarro water vapor variance < 0.00005	12.1%
9	Picarro water vapor variance > 0.5	0.6%
10	Picarro CO_2_ variance < 0.015	3.6%
11	Picarro CO_2_ variance > 25	1.7%
12	Picarro mean CO_2_ < 350 μmol mol^−1^	1.1%
13	Picarro CH_4_ variance > 0.00002	2.8%
14	Picarro CH_4_ variance < 0.0000007	5.1%

Flags are encoded as 14 bit values in binary notation, where for each bit, 1 = true and 0 = false for the criteria above. For convenience, flags are reported in both binary and decimal notation. For example, if *u* < 0.5 m s^−1^ (bit 2 = 1; binary = 00000000000010, decimal value = 2), sonic anemometer *T* variance > 2.5 (bit 6 = 1; binary value = 00000000100000, decimal value = 32), and Picarro CO_2_ variance < 0.015 (bit 10 = 1, binary value = 00001000000000, decimal value = 512), the quality flag descriptor would be reported as a binary value of 00001000100010, and a decimal value of 2 + 32 + 512 = 546. The “frequency” column reports the proportion of half-hourly periods (of ≈106,000 half-hourly periods in the 6-year data set reported here) receiving each quality flag. In total, slightly more than one-third (35.5%) of all half-hourly periods had a non-zero 14 bit quality flag.

In the filtered data set, we excluded turbulent fluxes if any relevant bit of the Howland QC flag was set to 1. We applied the flags assuming a hierarchy of flux measurements, i.e. if H was flagged, then LE was also flagged; if LE was flagged, then CO2_flux was also flagged; and if CO2_flux was flagged, then CH4_flux was also flagged. Thus bits 1 through 7 were applied to H, bits 1 through 9 were applied to LE, bits 1 through 12 were applied to CO2_flux, and bits 1 through and 14 were applied to CH4_flux. Additionally, if H was flagged, then all other measurements derived from the sonic anemometer—including sonic temperature, Tau, u*, and wind speed and direction—were also flagged. Flagged values were set to −9999 in the filtered data set.

We next used a simple empirically-based outlier detection method to identify the small number of remaining flux values that were statistically inconsistent with other measurements made under similar environmental conditions. To do this, we used a regression approach that accounted for covariation of environmental factors, and phenological effects associated with the time of year. We then calculated the interquartile range (IQR = *Q*_3_ − *Q*_1_, where *Q*_3_ and *Q*_1_ are the upper and lower quartiles, respectively) of the regression residuals, separately according to day vs. night and time of year. We conservatively excluded fluxes that were more than 6*IQR above *Q*_3_ or below *Q*_1_. Similar methods are commonly used in the literature, but a more aggressive threshold (e.g. 3*IQR) is typically used. Based on our previous work, we recognize that flux measurement errors have a leptokurtic distribution^36^, and large measurement errors are thus more likely than if errors followed a Gaussian distribution. Our goal was not complete “cleaning” of the data set, which might have resulted in discarding of valid measurements, but rather to identify the most extreme outliers. For H, 0.85% of all observations (910 of 106367 half-hours) were flagged as outliers, with 60% of those outliers occurring at night. For LE, 0.67% (710) of all observations were flagged as outliers, with 75% of those occurring at night. For CO_2__flux, 0.13% (140) of all observations were flagged as outliers, and for CH_4__flux, 0.14% (153) of all observations were flagged as outliers. Outliers were set to −9999 in the filtered data set.

We use the micrometeorological sign convention: flux into the ecosystem (e.g. photosynthetic CO_2_ uptake, CH_4_ consumption), is defined as a negative flux, whereas flux from the ecosystem to the atmosphere is a positive flux. Note that the flux units for both CO_2_ and CH_4_ are μmol m^−2^ s^−1^ in the unfiltered data set, but in the filtered data set—following the AmeriFlux convention—the flux units for CH_4_ are nmol m^−2^ s^−1^.

### Environmental measurements

We have for many years conducted measurements, from the main Howland tower, of key environmental and meteorological variables that are relevant to interpretation and modelling of ecosystem-atmosphere flux data. Like the tower fluxes, these data are reported at a 30-minute temporal resolution, which typically represents the mean of higher-frequency instantaneous measurements. For example, solar radiation measurements are taken every 15 s, but only the 30-minute mean is logged.

Environmental and meteorological measurements reported here include air temperature (shielded, ventilated platinum resistance thermometer), solar radiation (photosynthetic photon flux density, PPFD; model PAR lite quantum sensor, Kipp & Zonen, Delft, the Netherlands), net radiation (model CNR-4, Kipp & Zonen, Delft, the Netherlands), precipitation (heated tipping bucket rain gage, model TR-525; Texas Electronics, Dallas, TX, USA), and air pressure (model PTB100A analog barometer; Vaisala, Vantaa, Finland), all of which are measured at the top of the tower. Additionally, soil temperature at 10 cm depth (thermocouple) and water table depth (submersible pressure transducer model WL400; Global Water Instrumentation, College Station, TX, USA) have been measured 30 m from the base of the tower.

Through intercomparison of the PPFD, shortwave, and longwave radiation measurements at the main Howland tower (US-Ho1) together with simultaneous measurements made at the west Howland tower (US-Ho2; located 800 m away), and modeled clear-sky incident shortwave fluxes^37^, we screened the radiation data sets for extreme outliers, which could be attributed to instrument malfunction and snow on sensors. The number of half-hourly data points excluded in this way was generally very small, and in all cases well under 1% of the measured values (Table 3). Differences between incoming longwave radiation measured at the main and at the west tower (LW_IN_1_1_1 and LW_IN_2_1_1) are attributed to the lack of a heater/blower on the west tower instrument. A scatter plot of the outgoing shortwave radiation measurements at the main and west tower (SW_OUT_1_1_1 and SW_OUT_2_1_1) reveals an interesting nonlinear (“banana”) shape which implies some differences in surface reflectance as a function of solar elevation. The maximum measured difference between the two sensors is approximately 20 W m^−2^. Because the two shortwave sensors are on different towers, some differences are to be expected: the main tower is a walk-up tower, with a larger canopy hole, while the west tower is a triangular mast with a smaller canopy hole. The influence of the tower itself may be larger at the main tower. While the forest composition and structure is similar between the two towers, it is not identical. There may be differences in shadowing, canopy continuity and ground view (including snow on ground), and even the dominant species that are most prominent in the field of view of the instrument. Individually or together, these differences are likely sufficient to explain the observed difference in reflected shortwave radiation.

**Table 3:** Number of 30-minute radiation flux measurements (2012–2018), and the number of measurements flagged and removed through data screening based on sensor intercomparison.

Radiation flux	Number of measured values	Values removed through screening	Percentage of measured values removed
NETRAD_1_1_1	28,716	5	0.02%
NETRAD_2_1_1	99,423	618	0.62%
PPFD_IN_1_1_1	105,553	93	0.09%
SW_IN_1_1_1	28,762	28	0.10%
SW_OUT_1_1_1	28,754	20	0.07%
LW_IN_1_1_1	28,724	0	0.00%
LW_OUT_1_1_1	28,724	0	0.00%
SW_IN_2_1_1	99,502	276	0.28%
SW_OUT_2_1_1	99,503	277	0.28%
LW_IN_2_1_1	99,438	388	0.39%
LW_OUT_2_1_1	99,438	388	0.39%

Screening was designed to eliminate only the most obvious outliers, associated with snow or instrument malfunction.

### Chamber measurements

An automated, chamber-based system was used to quantify soil CO_2_, CH_4_ and N_2_O fluxes within the footprint of the main Howland tower (Fig. 1). The system, and details of sampling methods and data processing, are described in detail in previous publications^27,28^. Briefly, automated chambers (each 30.5 cm in diameter; between measurements the chamber top was lifted, using a pneumatic piston, off a PVC collar permanently inserted into the soil surface) were installed in one of three topographic positions, (1) upland: forest-dominated, and characterized by well-drained soils; (2) transitional: sphagnum-dominated, and characterized by sporadic inundation; and (3) wetland: sphagnum-dominated, underlain by peat deposits approximately 1 m deep, and characterized by continuous inundation. (The upland plots were also the site of a trenching experiment that was initiated in late fall of 2012 to permit partitioning of soil respiration to autotrophic and heterotrophic components^38^. Root exclusion trenches 1 m deep were dug around three 5 m × 5 m plots; the trenches were then lined with plastic sheeting and backfilled. One automated chamber was placed in each of the trenched plots and three chambers were left in their original upland positions as controls. Measurement of the trenched plots occurred from 2012–2015, and these data are included here).

Where the chambers were installed, and what trace gas fluxes were measured, varied among years. Deployments are summarized by year and topographic position in Table 4, with chamber specifics in Table 5. Because of differences among years in the measurement objectives and number of chambers deployed, the exact frequency at which a specific chamber was sampled may have varied over time.

**Table 4:** Summary of chambers deployed for measurement of soil greenhouse gas fluxes at Howland Forest, 2012–2016, by soil drainage class and treatment (if applicable).

Year	Drainage/treatment			
	Upland, control	Upland, trenched	Transitional	Wetland
2012	3 (CO_2_, CH_4_); CRDS	3 (CO_2_, CH_4_); CRDS	2 (CO_2_, CH_4_); CRDS	None
2013	3 (CO_2_, CH_4_); CRDS	3 (CO_2_, CH_4_); CRDS	None	5 (CO_2_, CH_4_,N_2_O); IRGA, QCL
2014	3 (CO_2_, CH_4_); CRDS	3 (CO_2_, CH_4_); CRDS	3(CO_2_, CH_4_); CRDS	3 (CO_2_, CH_4_,N_2_O); IRGA, QCL
2015	5 (CO_2_, CH_4_,N_2_O); IRGA, QCL	3 (CO_2_, CH_4_,N_2_O); IRGA, QCL	3 (CO_2_, CH_4_,N_2_O); IRGA, QCL	3 (CO_2_, CH_4_,N_2_O); IRGA, QCL
2016	4 (CO_2_, CH_4_,N_2_O); IRGA, QCL	None	5 (CO_2_, CH_4_,N_2_O); IRGA, QCL	3 (CO_2_, CH_4_,N_2_O); IRGA, QCL

Each cell reports the number of chambers installed, the trace gas fluxes measured, and the instrument used to measure trace gas concentrations. CRDS = cavity ring-down spectrometer (Picarro model G2121-i); QCL = quantum cascade laser (Aerodyne Research model TILDAS CS S/N #24); IRGA = infrared gas analyser (LI-COR model 6252).

**Table 5:** Details of chambers deployed for measurement of soil greenhouse gas fluxes at Howland Forest, 2012–2016.

Chamber_ID	Year installed	Year removed	Drainage/treatment	AmeriFlux code
Chamber 2	2012	2016	Upland, control	CMB_FC_2_1_1
Chamber 4	2012	2016	Upland, control	CMB_FC_4_1_1
Chamber 6	2012	2016	Upland, control	CMB_FC_6_1_1
Chamber 7	2012, 2014	2015	Transitional	CMB_FC_7_1_1
Chamber 8	2012, 2014	2016	Transitional	CMB_FC_8_1_1
Chamber 9	2014	2016	Transitional	CMB_FC_9_1_1
Chamber 10	2013	2016	Wetland	CMB_FC_10_1_1
Chamber 11	2013	2016	Wetland	CMB_FC_11_1_1
Chamber 12	2013	2016	Wetland	CMB_FC_12_1_1
Chamber 13	2015	2015	Upland, control	CMB_FC_13_1_1
Chamber 14	2015	2015	Upland, control	CMB_FC_14_1_1
Chamber 15	2013	2013	Wetland	CMB_FC_15_1_1
Chamber 16	2013	2013	Wetland	CMB_FC_16_1_1
Chamber 17	2016	2016	Transitional	CMB_FC_17_1_1
Chamber 18	2016	2016	Transitional	CMB_FC_18_1_1
Chamber 19	2016	2016	Transitional	CMB_FC_19_1_1
Chamber 20	2012	2015	Upland, trenched	CMB_FC_20_1_1
Chamber 21	2012	2015	Upland, trenched	CMB_FC_21_1_1
Chamber 22	2012	2015	Upland, trenched	CMB_FC_22_1_1
Chamber 23	2016	2016	Upland, control	CMB_FC_23_1_1

For each chamber ID, the corresponding years of installation, location of installation (soil drainage class and treatment, if applicable), and AmeriFlux code are given. Note that only chambers 2, 4, and 6 were installed continuously, at the same locations, during the 4-year measurement period covered by this dataset (2012–2016).

From 2012 to 2016, soil fluxes from each chamber were measured approximately once per hour, 24 h per day, during the snow-free period when vegetation was active (May to November). Different gas analysers were deployed depending on the measurement objectives. To measure soil CO_2_ fluxes, we used an infrared gas analyser (model 6252; LI-COR Biosciences, Lincoln, NE, USA); to measure soil CO_2_ and CH_4_ fluxes, we used a cavity ring-down spectrometer (model G2121-i; Picarro Inc., Santa Clara, CA, USA); to measure soil CH_4_ and N_2_O fluxes, we used a quantum cascade laser (TILDAS CS, Aerodyne Research Inc., Billerica, MA, USA). To measure soil CO_2_, CH_4_, and N_2_O fluxes, we used the infrared gas analyser and the quantum cascade laser in series^28^.

Trace gas fluxes were determined using chamber headspace concentrations measured (1 Hz) over a 4-minute period, beginning 60 s and ending 300 s after the chamber top closed. Thus, each measurement sequence required 5 minutes. We note that noise in the 1 Hz concentration data output by the analyser will propagate directly to uncertainty in the calculated flux, particularly when the flux is small and the noise is relatively large in comparison to the change in headspace concentration. Thus, CH_4_ fluxes calculated from the quantum cascade laser measurements have better precision than fluxes calculated from the cavity ring-down spectrometer, and CO_2_ fluxes have better precision than either the CH_4_ or N_2_O fluxes.

Fluxes were calculated from the linear regression of change in headspace concentration over time and were scaled up from the collar area, corrected for atmospheric pressure and temperature. Units for the fluxes are as follows: CO_2_ flux, μmol CO_2_ m^−2^ s^−1^ ( = 43.2 mg C-CO_2_ m^−2^ hr^−1^); CH_4_ flux, nmol CH_4_ m^−2^ s^−1^ ( = 43.2 μg C-CH_4_ m^−2^ hr^−1^); and N_2_O flux, nmol N_2_O m^−2^ s^−1^ ( = 100.8 µg N-N_2_O m^−2^ hr^−1^.

At upland sites, soil moisture (volumetric water content, cm^3^ H_2_O cm^−3^ soil volume; measured with a model CS-616 water content reflectometer, calibrated to site soil conditions; Campbell Scientific, Logan, UT, USA) and soil temperature (°C, measured with a Type T thermocouple) were logged continuously from 2012 through 2016.

Following our standard soil respiration QC procedures^27^, measured CO_2_ fluxes were excluded if the correlation between headspace CO_2_ concentration and time was insufficiently high (R^2^ < 0.9), on the assumption that a poor correlation (nonlinear or noisy) likely indicates that the chamber lid did not close properly. All soil fluxes have been filtered to remove data obtained when the measurement system was compromised, e.g. power or instrument failure, and during periods of instrument calibration or testing. As with the eddy covariance measurements, our sign convention is that a negative flux indicates uptake by the soil (i.e., CH_4_ consumption is a negative flux), and a positive flux indicates emission from the soil (i.e., respiration of CO_2_ is a positive flux).

## Data Records

The data set presented here, which is available within Figshare^29^ and released under a CC-BY 4.0 license, consists of (1) the “tower flux” data files, which includes three files derived from our primary gas analyzer (Picarro CRDS) as well a fourth file derived from our secondary (backup) gas analyzer (LI-COR IRGA); (2) a “chamber flux” data file; and (3) several additional metadata files. The tower flux and chamber flux data files are formatted as comma-delimited ASCII text. Missing values are denoted as −9999.

### Tower fluxes

The “tower flux” data files contain continuous measurements of the ecosystem-atmosphere energy (H and LE) and trace gas (CO2_flux and CH4_flux) fluxes, reported at a 30-minute time step, covering the period June 2012 through June 2018. These files also include derived quantities including uncertainties, quality control flags, and flux footprint estimates, as well as basic environmental and meteorological data.

For ease of use, we have divided the tower flux data into four separate files, as follows:

1. **Unfiltered EddyPro output**. This file contains the processed but unfiltered tower fluxes (calculated using data from the Picarro CRDS), as output by the EddyPro software at a 30 minute time-step, as well as the associated enviro-meteorological data, and is named US-Ho1_HH_201206060000_201806302330_EP.csv. The columns of this data file are described in Online-only Table 1. This file is distributed through Figshare as it contains numerous columns that at present cannot be distributed via AmeriFlux. This includes variances and covariances, flux uncertainties, spectral correction factors, and trace gas time lags that may be of interest to some data users. Additionally, this file has not been filtered using the standard Howland QC flags, providing the data user the opportunity to apply their own filtering methods (e.g. Mauder and Foken^39^ QC flags; see Usage Notes, below) if desired.
2. **QC and Outlier Flags**. This file contains the standard Howland QC flags (Table 2), reported as both decimal and binary values, and summarized for each turbulent flux (H_qc, LE_qc, CO2_flux_qc, CH4_flux_qc), where a value of 1 is used to indicate a measurement that does not pass our QC criteria. This file also contains a summary flag for each half-hour turbulent flux measurement (H_flag, LE_flag, CO2_flux_flag, CH4_flux_flag), which integrates the QC and outlier flags as follows: 0, valid measurement; 1, missing or fails standard Howland QC criteria; 2, outlier more than 6*IQR below *Q*_1_; and 3, outlier more than 6*IQR above *Q*_3_. The summary flag has been applied to the turbulent fluxes reported in the filtered flux file; measured fluxes are reported if the summary flag equals zero, and are set to −9999 if the summary flag equals 1, 2 or 3. Additionally, a summary flag value of 4 is used to indicate suspect nocturnal data (based on a u* threshold; see Usage Notes), although following AmeriFlux data standards we have ***not*** removed these measurements from the filtered data set. The QC and outlier flags file is named US-Ho1_HH_201206060000_201806302330_QC.csv. The columns of this data file are described in Table 6. This file is distributed through Figshare as the data it contains cannot be distributed via AmeriFlux.

**Table 6:** Headings and description of data columns in the QC and outlier flags file for the Howland tower flux data set (US-Ho1_HH_201206060000_201806302330_QC.csv).

Label (units or format)	Description
TIMESTAMP_START (YYYYMMDDHHMM)	AmeriFlux-format time stamp at beginning of 30-minute averaging period
TIMESTAMP_END (YYYYMMDDHHMM)	AmeriFlux-format time stamp at end of 30-minute averaging period
HowQC_bit14 … HowQC_bit1	Standard Howland QC flags for each of the 14 criteria listed in Table 2. Zero values indicate good data.
HowQC_Dec	Sum of Howland QC flags (bits 14 through 1), in decimal notation
HowQC_Bin	Sum of Howland QC flags, expressed in 14-bit binary notation.
H_HowQC	Howland QC flag for H and other quantities derived from sonic anemometer, set to 0 (good data) if the sum of QC flag bits 1 through 7 equals 0, and 1 otherwise (bad data).
LE_HowQC	Howland QC flag for LE, calculated based on the sum of QC flag bits 1 through 9
CO2_flux_HowQC	Howland QC flag for CO2_flux, calculated based on the sum of QC flag bits 1 through 12
CH4_flux_HowQC	Howland QC flag for CH42_flux, calculated based on the sum of QC flag bits 1 through 14
H_flag, LE_flag, CO2_flux_flag, CH4_flux_flag	Summary flag for filtering H, LE, CO2_flux and CH4_flux; 0 = valid measurement, 1 = missing or fails standard Howland QC criteria, 2 = outlier more than 6*IQR below *Q*_1_, and 3 = outlier more than 6*IQR above *Q*_3_. Additionally, a value of 4 is used to indicate night-time data recorded under periods of high atmospheric stability and low turbulence (PPFD ≤ 5 μmol m^−2^ s^−1^ and u* ≤ 0.25 m s^−1^)
3. **Filtered half-hourly AmeriFlux-format dataset**. This file contains the filtered tower fluxes, as well as associated enviro-meteorological data, at a 30 minute time step, formatted according to AmeriFlux standards. Following AmeriFlux naming conventions, the AmeriFlux-format tower fluxes dataset is named US-Ho1_HH_201206060000_201806302330.csv. The columns of this file are described in Table 7. This file is distributed through Figshare, and an identical file has been uploaded to the AmeriFlux data archive, where it has undergone the standard AmeriFlux checks for data quality and consistency, and where it is available as part of the larger US-Ho1 data record (since 1996)^30^.

**Table 7:** Headings and description of data columns in the filtered AmeriFlux-format dataset for the Howland tower (US-Ho1_HH_201206060000_201806302330.csv).

Label (units or format)	EddyPro label	Description
TIMESTAMP_START (YYYYMMDDHHMM)		AmeriFlux-format time stamp at beginning of 30-minute averaging period
TIMESTAMP_END (YYYYMMDDHHMM)		AmeriFlux-format time stamp at end of 30-minute averaging period.
FC_1_1_1 (μmol m^−2^ s^−1^)	CO2_flux	CO_2_ flux, filtered according to Table 6
FC_SSITC_TEST_1_1_1	qc_CO2_flux	Mauder and Foken QC flag for CO_2_ flux
CO2_1_1_1 (µmol mol^−1^)	CO2_mole_fraction	Mole fraction of CO_2_ (instrument: Picarro G2311-f)
H_1_1_1 (W m^−2^)	H	Sensible heat flux, H, filtered according to Table 6
H_SSITC_TEST_1_1_1	qc_H	Mauder and Foken QC flag for H
LE_1_1_1 (W m^−2^)	LE	Latent heat flux, LE, filtered according to Table 6
LE_SSITC_TEST_1_1_1	qc_LE	Mauder and Foken QC flag for LE
H2O_1_1_1 (µmol mol^−1^)	H2O_mole_fraction	Mole fraction of H_2_O (instrument: Picarro G2311-f)
NEE_1_1_1 (μmol m^−2^ s^−1^)		Net ecosystem exchange of CO_2_, calculated as CO_2_ turbulent flux plus CO_2_ storage flux
FCH4_1_1_1 (nmol m^−2^ s^−1^)	CH4_flux	CH_4_ flux, filtered according to Table 6. Note: *AmeriFlux units differ from EddyPro output units*
FCH4_SSITC_TEST_1_1_1	qc_CH4_flux	Mauder and Foken QC flag for CH_4_
CH4_1_1_1 (nmol mol^−1^)	CH4_mole_fraction	Mole fraction of CH_4_. Note: *AmeriFlux units differ from EddyPro output units* (instrument: Picarro G2311-f)
RH_EP_1_1_1 (%)	RH	Ambient relative humidity
VPD_EP_1_1_1 (hPa)	VPD	Ambient water vapor pressure deficit. Note: *AmeriFlux units differ from EddyPro output units*
T_SONIC_1_1_1 (°C)	sonic_temperature	Mean temperature of ambient air, as measured by the sonic anemometer. Note: *AmeriFlux units differ from EddyPro output units*
TAU_1_1_1 (kg m^−1^ s^−1^)	Tau	Momentum flux
USTAR_1_1_1 (m s^−1^)	u*	Friction velocity
WD_1_1_1 (degrees)	wind_dir	Direction from which the wind blows (instrument: ATI SAT-211/3 K)
WS_1_1_1 (m s^−1^)	wind_speed	Mean wind speed (instrument: ATI SAT-211/3 K)
ZL_1_1_1	(z-d)/L	Monin-Obukhov stability parameter
FETCH_70_1_1_1 (m)	x_70%	Along-wind distance providing 70% (cumulative) contribution to turbulent fluxes
FETCH_90_1_1_1 (m)	x_90%	Along-wind distance providing 90% (cumulative) contribution to turbulent fluxes
FETCH_MAX_1_1_1 (m)	x_peak	Along-wind distance providing the highest (peak) contribution to turbulent fluxes
PA_1_1_1 (kPa)	air_pressure	Mean pressure of ambient air, as measured by the gas analyzer. Note: *AmeriFlux units differ from EddyPro output units*
TA_1_1_1 (°C)	air_temperature	Mean temperature of ambient air, as measured by platinum resistance thermometer. Note: *AmeriFlux units differ from EddyPro output units*
P_RAIN_1_1_1 (mm)		Precipitation falling as rain, as measured with a tipping bucket rain gage which is heated in winter but which may under-estimate precipitation falling as snow (instrument: Texas Electronics TR-525M)
P_2_1_1 (mm)		Total precipitation (liquid + solid) measured by U.S. Climate Reference Network data (hourly converted to half hour) from Old Town, ME, Roger’s Farm site (~40 km from US-Ho1), WBAN # 94644
NETRAD_1_1_1 (W m^−2^)		Net radiation, measured by sensor 1 (Instrument: Kipp & Zonen CNR-4, mounted on the same tower)
NETRAD_2_1_1 (W m^−2^)		Net radiation, measured by sensor 2 (instrument: Kipp & Zonen CNR-1, mounted on a tower in similar vegetation 800 m to the NW)
PPFD_IN_1_1_1 (μmol m^−2^ s^−1^)		Incident photosynthetic photon flux density (instrument: Li-Cor Li-190SA, mounted on the same tower)
SW_IN_1_1_1 (W m^−2^)		Incident shortwave radiation, measured by sensor 1
SW_OUT_1_1_1 (W m^−2^)		Reflected shortwave radiation, measured by sensor 1
LW_IN_1_1_1 (W m^−2^)		Downwelling longwave radiation, measured by sensor 1
LW_OUT_1_1_1 (W m^−2^)		Upwelling longwave radiation, measured by sensor 1
SW_IN_2_1_1 (W m^−2^)		Incident shortwave radiation, measured by sensor 2
SW_OUT_2_1_1 (W m^−2^)		Reflected shortwave radiation, measured by sensor 2
LW_IN_2_1_1 (W m^−2^)		Downwelling longwave radiation, measured by sensor 2
LW_OUT_2_1_1 (W m^−2^)		Upwelling longwave radiation, measured by sensor 2
TS_1_1_1 (°C)		Soil temperature at 5 cm depth (instrument: Type T thermocouple)
WTD_1_1_1 (cm)		Water table depth, negative values indicate water table below soil surface (shallow well) (instrument: Global Water WL400)
WTD_2_1_1 (cm)		Water table depth, negative values indicate water table below soil surface (deep well) (instrument: Global Water WL400)

Column headings follow standard AmeriFlux naming conventions.
4. **Unfiltered EddyPro output for a second gas analyser**. This file contains the processed but unfiltered tower fluxes (calculated using data from the LI-COR Li-7200 IRGA; note that this instrument does not measure CH_4_), as output by the EddyPro software at a 30 minute time-step, and is named US-Ho1_HH_201206060000_201806302330_EP LI-COR.csv. The columns of this data file are described in Online-only Table 1. This dataset is only distributed through Figshare; fluxes calculated from the LI-COR analyser have not been uploaded to AmeriFlux because of concerns about system performance in 2018. Fluxes from this data file were used in the technical validation analyses described below.

### Chamber fluxes

The “chamber flux” data file contains measurements of soil fluxes of CO_2_, CH_4_, and N_2_O, with measurements from each chamber reported approximately hourly during the growing season (2012–2016). The chamber fluxes file is named US-Ho1_CMB_201201010000_201701010000.csv, and the columns of the data file are described in Table 8. This file is distributed through Figshare. An identical file has been uploaded to the AmeriFlux archive, but it contains data that cannot be distributed through AmeriFlux.

**Table 8:** Headings and description of data columns in the Howland soil chamber flux dataset (US-Ho1_CMB_201201010000_201701010000.csv).

Label (units or format)	Description
Timestamp_Start (YYYYMMDDHHMM)	AmeriFlux-format time stamp at beginning of 30-minute period
Timestamp_End (YYYYMMDDHHMM)	AmeriFlux-format time stamp at end of 30-minute period
CMB_TIME_START_x_1_1	Time stamp at beginning of 5-minute measurement period for chamber *x*, where *x* = 2, 4, 6 … 23.
CMB_TIME_END_x_1_1	Time stamp at end of 5-minute measurement period for chamber *x*
CMB_FC_x_1_1 (μmol CO_2_ m^−2^ s^−1^)	Measured CO_2_ flux for chamber *x*
CMB_FCH4_x_1_1 (nmol CH_4_ m^−2^ s^−1^)	Measured CH_4_ flux for chamber *x*
CMB_FN2O_x_1_1 (nmol N_2_O m^−2^ s^−1^)	Measured N_2_O flux for chamber *x*
…	Data for additional chambers
CMB_SWC_x_1_1 (% vol.)	Measured soil water content for chamber *x*, where *x* = 2, 4, 6, 20, 21, 21
…	Data for additional chambers
CMB_TS_x_1_1 (°C)	Measured soil temperature for chamber *x*, where *x* = 2, 4, 6, 20, 21, or 21
…	Data for additional chambers

### Additional files

The configuration and metadata files used for the Eddy Pro processing described here (files: processing_2018-11-21T083742_adv.eddypro and main2012–2018.metadata), as well as the AmeriFlux-format machine-readable Biological, Ancillary, Disturbance and Metadata (BADM) template (which contains information about the site, including standing biomass, leaf area index, and soil chemistry) (file: AMF_US-Ho1_BIF_LATEST 04 09 2019.xlsx) and the Instrument Operations template (which contains information about when specific instruments were installed or removed) (file: 2019-US-Ho1_ Instrument_Ops 03 14 2019.xlsx), are also archived on Figshare^29^.

## Technical Validation

### Site overview

The forest in the vicinity of the main Howland tower is nearly ideal from the perspective of making tower-based flux measurements over tall vegetation; forest cover is extensive and homogeneous, and the topography is generally flat^13^. As one of the longest-running AmeriFlux sites, the eddy covariance flux measurements at Howland have been carefully scrutinized over the last two decades. For example, the environmental and flux measurements from the main Howland tower have been regularly evaluated against data recorded by the AmeriFlux Portable Eddy Covariance System, which was most recently deployed adjacent to our own instrumentation for a 10-day period in the summer of 2016. Additionally, since 1998 environmental and flux measurements have also been conducted at the “west” Howland tower, located about 800 m to the north-west of the main tower, in an extensive forest stand with composition and structure similar to that surrounding the main tower. Analysis of the coherence spectra for environmental variables and fluxes recorded on the two towers has shown excellent agreement between the two measurement systems over time scales of hours to days, while at the annual time step, the net ecosystem exchange of CO_2_ measured at the two towers was found to differ by less than 6%^12^. These analyses point to the high quality of eddy covariance flux measurements at Howland Forest, and the representativeness of the main tower in relation to the immediately surrounding landscape. We note also that data from the main and west towers were used to develop a novel method of assessing the random uncertainty in 30-minute CO_2_, H_2_O and energy fluxes^12,40^, which has then been applied to estimate uncertainties in annual ecosystem C budgets^41,42^. Thus, in general the eddy covariance fluxes measured at Howland are known to be of high quality, with well-characterized uncertainties.

Here we conduct three additional analyses to further assess the technical quality of the tower-based measurements. First, we compare LE and CO_2_ fluxes calculated using H_2_O and CO_2_ concentrations measured with our Picarro CRDS against those calculated using concentrations measured simultaneously with a LI-COR IRGA. Second, we compare the long-term patterns in CH_4_ concentration measured with our analyser against independent atmospheric CH_4_ concentration measurements from two climate monitoring observatory stations. Finally, we conduct an analysis of the quality control flags and estimated random uncertainties in the CH_4_ flux measurements.

### Comparison of fluxes calculated using independent CO_2_ mixing ratio measurements

**Fig. 3:**
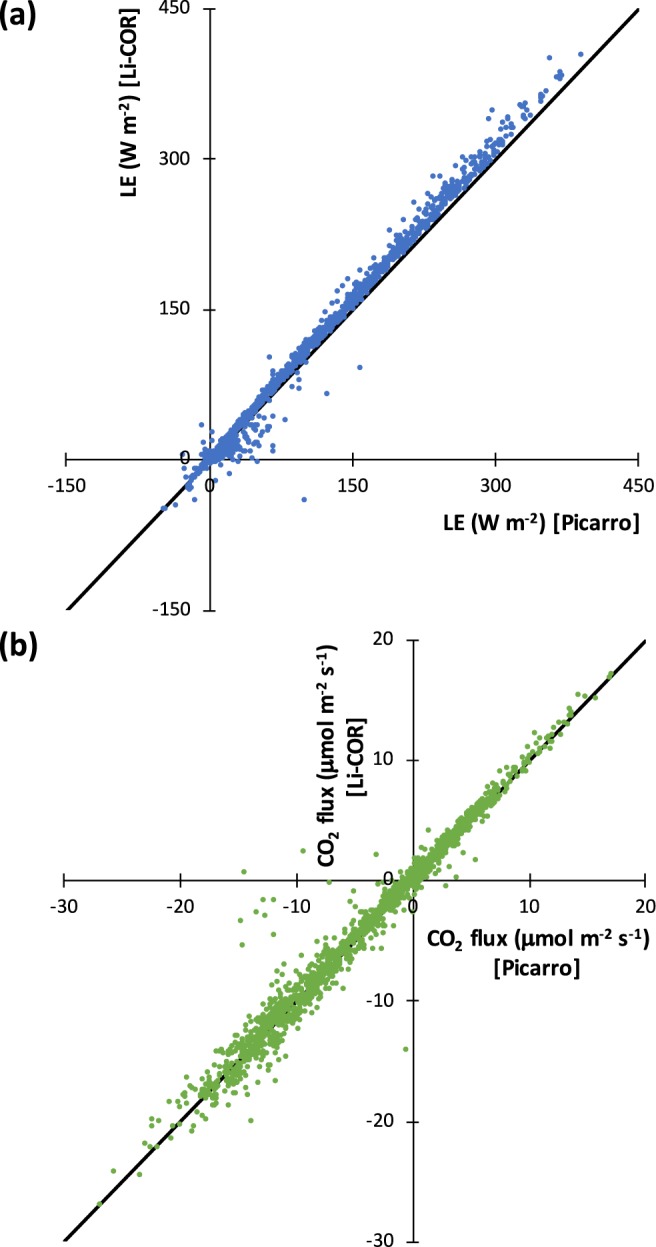
Comparison of ecosystem-atmosphere fluxes calculated using trace gas concentrations measured with two different gas analyzers (Picarro CRDS and LI-COR IRGA), but orthogonal wind components from a single sonic anemometer. (**a**) Latent heat flux (LE), (**b**) CO_2_ flux. Independent air samples were measured by each analyzer, although gas inlets were located adjacent to each other at the top of the main Howland (US-Ho1) tower. Data were recorded at 5 Hz. 30-minute fluxes were calculated using the eddy covariance method. Data are from 2012. The standard Howland QC filtering, including a nocturnal u* threshold (excluding flux measurements when u* ≤ 0.25 m s^−1^), was applied to both data sets. Black diagonal lines indicate 1:1.

Since we installed the Picarro CRDS at Howland in 2012, we have operated it in parallel with a co-deployed fast response closed-path CO_2_/H_2_O infrared gas analyser (IRGA model Li-7200, Li-Cor Inc., Lincoln, NE) for redundancy and quality assurance. (Prior to 2012, we exclusively used closed-path LI-COR IRGAs for flux measurements on the main Howland tower.) The two instruments have independent air sampling systems (tubing, pump, and flow control), although air inlets are located adjacent to each other at the top of the tower. For flux calculations, orthogonal wind components from a single sonic anemometer are used in conjunction with the H_2_O and CO_2_ concentrations (for CO_2_, dry air mole fraction) data reported from each analyser. The level of agreement between the fluxes calculated from these two systems (see Fig. 3 for a comparison using 2012 data; see Table 9 for statistics for all years 2012–2018; see also ref.^16^) gives us confidence in the overall quality of the fluxes (specifically LE and CO_2__flux, and by extension CH_4__flux) measured using the Picarro CRDS. While the agreement between the two analyzers is not as good in 2018 compared to the previous years, we attribute this to known issues with the LI-COR-based system in that year, including analyser calibration and pump/flow controller problems which do not affect the Picarro measurements.

**Table 9:** Correlation and regression statistics, by year, for agreement between ecosystem-atmosphere fluxes calculated using trace gas concentrations measured with two different gas analyzers (Picarro CRDS and LI-COR IRGA).

Flux	Year	2012	2013	2014	2015	2016	2017	2018
Latent heat	*N*	1916	8336	7776	8879	8566	5931	4069
	Correlation	0.997	0.986	0.976	0.981	0.977	0.978	0.942
	Slope	1.06	1.02	0.98	1.00	0.98	1.08	1.11
	Intercept	−1.18	−2.91	−3.18	−1.09	−2.33	3.40	3.25
CO_2_	*N*	1933	8205	7644	8928	8600	5937	4070
	Correlation	0.987	0.988	0.985	0.977	0.975	0.992	0.979
	Slope	0.99	1.00	0.99	0.99	0.98	0.96	1.20
	Intercept	0.01	0.03	0.00	0.03	0.04	0.01	0.21

Units are W m^−2^ for latent heat flux (LE), and μmol m^−2^ s^−1^ for CO_2_ flux. *N* is the number of half-hourly measurements included in the comparison; correlation is Pearson’s *r*; slope and intercept are least-squares regression statistics (*y* = LI-COR flux; *x* = Picarro flux).

### Long-term assessment of CH_4_ analyser performance

For Howland, we calculated the monthly mean CH_4_ concentration (dry air mole fraction) from the 30-minute mid-day (10 am to 2 pm, local standard time) mean values. From the approximately 18,000 mid-day half-hourly data points recorded between 2012 and 2018, we excluded from the calculation 323 half-hourly measurements where the measured CH_4_ concentration was greater than 2600 ppb (61% of these high measurements occurred during a brief period late in 2014), and 8 half-hourly measurements where the measured CH_4_ concentration was less than 1500 ppb. There were 1075 missing data points when the CH_4_ concentration was not recorded due to power or instrument failure. Within each monthly period, the standard deviation of the mean half-hourly CH_4_ concentrations had a mean value of 20 ppb, and with ≈240 measurements averaged each month, the standard error of the mean was in almost all cases less than 2 ppb. The monthly median tended to be somewhat lower (by 4 ± 3 ppb) than the monthly mean, but the temporal patterns were essentially identical.

**Fig. 4:**
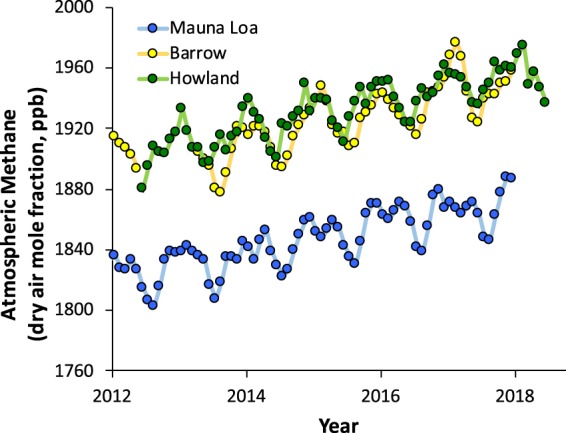
Seasonal variation and long-term trend in atmospheric CH_4_. Howland data represent the monthly mean CH_4_ concentration, calculated across mid-day (10 am to 2 pm, local standard time) values, after outlier removal (CH_4_ < 1500 ppb, or CH_4_ > 2600 ppb). Mauna Loa and Barrow data are courtesy of NOAA^43,44^.

In Fig. 4, we compare the Howland (Maine) data with data from Mauna Loa (Hawaii) and Barrow (Alaska), where ongoing long-term atmospheric CH_4_ concentration measurements are maintained by researchers from the National Oceanic and Atmospheric Administration (NOAA)^43,44^. For the NOAA data, sub-hourly measurements have been similarly filtered for outliers (<1500 ppb or >2600 ppb), averaged to hourly values, and then screened to distinguish samples of regionally representative air. These are then filtered using a rule-based editing algorithm to exclude measurements obtained when the analytical instrument was not working properly. The NOAA instruments (an automated gas chromatograph using flame ionization detection at Mauna Loa, and since 2013 a laser-based optical analyser at Barrow) are regularly calibrated against reference standards.

Overall, the monthly mean CH_4_ concentrations from Howland show two obvious features. First, there is a pronounced seasonal cycle, with CH_4_ varying by 30–40 ppb between a summertime minimum and wintertime maximum. Second, there is clear rising trend, with CH_4_ increasing at a rate of almost 10 ppb per year, from an annual mean of just under 1910 ppb at the start of our measurement record to almost 1960 ppb at the end of our record. The excellent agreement between the Howland CH_4_ measurements and the NOAA measurements—particularly for Barrow—demonstrates the long-term calibration of our instrument (specifically, the lack of calibration drift and hence the overall accuracy). Together with precision statistics reported by the manufacturer, this gives us confidence in the sustained quality of our CH_4_ flux measurements. There is no evidence of degraded instrument performance over the six years of measurements.

### Assessment of quality control flags and random uncertainty in tower CH_4_ fluxes

Across the more than 100,000 half-hourly periods covered by the eddy covariance dataset, there were missing CH_4_ fluxes (due to power or instrument failure) only 8% of the time. A further 15% were assigned a Mauder and Foken^39^ (M&F) QC flag of 2, indicating low quality measurements. Therefore, more than 75% of the time the fluxes were considered to be of “usable” quality, with 37% receiving an M&F QC flag of 0 (the highest quality) and 41% an M&F QC flag of 1.

Within EddyPro, the method of Finkelstein and Sims^45^ was used to estimate random uncertainties in all calculated fluxes. Because the CH_4_ fluxes measured at Howland are generally small, an important question is whether we are measuring signal (i.e. exceeding the detection limit for a measurable flux) or noise. If the ratio of the measured flux to the uncertainty has an absolute value greater than 2, then the measured flux can be considered significantly different from zero with high (95%) confidence. For CH_4_ fluxes with an M&F QC flag of 0, the median uncertainty ratio was 2.4; 65% of the time the uncertainty ratio was greater than 2, and 32% of the time it was greater than 3. For CH_4_ fluxes with an M&F QC flag of 1, the median uncertainty ratio was 1.9; 46% of the time the uncertainty ratio was greater than 2, and 22% of the time it was greater than 3. Thus, although CH_4_ fluxes measured at Howland tend to be small in magnitude, they are commonly above the detection limit of the eddy covariance method.

The above three analyses indicate the overall quality and technical validity of the tower-based fluxes that we report here.

### Automated chamber measurements

Uncertainties in our chamber-based soil flux measurement system have been assessed and quantified in several previous publications, which focused on the measurement of soil CO_2_ efflux^27,46^. Indeed, based on work at Howland, we have previously concluded that “[w]hile … potential sources of measurement error and sampling biases must be carefully considered, properly designed and deployed chambers provide a reliable means of accurately measuring soil respiration in terrestrial ecosystems”^47^.

We have also published a detailed quality assessment of the uncertainties in CH_4_ and N_2_O fluxes measured with our chamber system using the quantum cascade laser^28^. This analysis showed that the response time of the analyser was sufficiently fast, and sensitivity was sufficiently high, that we could measure fluxes quickly enough so as not to influence soil concentration gradients. Furthermore, we determined the minimum detectable fluxes using the method of Verchot *et al*.^48^; for the automated chamber system deployed at Howland these were estimated to be very low: ± 0.12 μg CH_4_-C m^−2^ h^−1^ (=0.0028 nmol CH_4_ m^−2^ s^−1^) and ±0.05 μg N_2_O-N m^−2^ h^−1^ (0.000496 nmols N_2_O m^−2^ s^−1^). Detection of such small fluxes is possible because of the high precision of the QCL instrument.

This previous work gives us high confidence in the overall quality of the soil fluxes of CO_2_, CH_4_, and N_2_O reported here.

## Usage Notes

### Recommended filtering criteria

The AmeriFlux-formatted data file included here has been filtered according to standard methods used for over two decades at Howland Forest, and is the data set recommended for most analyses. However, The Mauder and Foken^39^ QC flags included in the EddyPro output could alternatively be used for data filtering (avoiding values flagged as “2”).

At Howland, we have always adopted the friction velocity (“u* filtering”) method of removing night-time data recorded under periods of high atmospheric stability and low turbulence^12^. We therefore recommend that data from nocturnal periods (PPFD ≤ 5 μmol m^−2^ s^−1^) be excluded when u* ≤ 0.25 m s^−1^. These periods are indicated by a summary flag value of 4 in the QC and outlier flags file.

### Gap filling and flux partitioning

A variety of methods are commonly used to fill gaps in meteorological and flux data sets so that annual averages or integrals can be estimated^49^. However, following standard AmeriFlux protocols, the data here have not been gap-filled. For gap-filling of meteorological data sets, methods based on reanalysis products have been developed^50^ and these may be preferred to empirical methods based on mean diurnal variation. For gap-filling of CO_2_, H, and LE fluxes, the online gap-filling tool provided by the Max Planck Institute can be used (https://www.bgc-jena.mpg.de/bgi/index.php/Services/REddyProcWeb). This tool can also partition net fluxes to their underlying component fluxes, e.g. net CO_2_ flux is partitioned to ecosystem respiration and gross primary production^51,52^, which is valuable for ecosystem C budget analyses. A variety of methods (including temperature relationships, neural networks, linear interpolation, mean diurnal variation, etc.) have been used for gap-filling of CH_4_ fluxes^53^, but we are not aware of a consensus method.

### Complementary data sets

Long-term data from the Howland AmeriFlux site are available through the AmeriFlux data portal (https://ameriflux.lbl.gov/sites/site-search/#keyword=Howland). This includes CO_2_, H_2_O, and energy fluxes measured via eddy covariance, as well as meteorological and environmental data at a 30 minute time step. Measurements have been conducted at the main Howland tower (AmeriFlux site US-Ho1)^30^ since 1996 (the full US-Ho1 dataset available for download from AmeriFlux includes the filtered half-hourly AmeriFlux-format dataset described here); at the west Howland tower (AmeriFlux site US-Ho2)^54^, the site of a low-level N addition experiment, since 1998; and the east Howland tower (AmeriFlux site US-Ho3)^55^, the site of a shelterwood harvest experiment, since 2001.

Additional publicly-available data sets for Howland include the following:

1. Forest composition and biomass measurements were conducted as part of the NACP (North American Carbon Program) field campaign in 2009 and 2010 (https://daac.ornl.gov/cgi-bin/dsviewer.pl?ds_id=1046)^56^;
2. The AirMOSS (Airborne Microwave Observatory of Subcanopy and Subsurface) radar instrument provides high-resolution data on root-zone soil moisture, with periodic flights conducted over Howland between October 2012 and December 2015 (https://daac.ornl.gov/AIRMOSS/guides/AirMOSS_L1_Sigma0_Howlnd.html)^57^;
3. COSMOS (Cosmic-ray Soil Moisture Observing System) probes deployed at Howland use cosmogenic neutrons to derive an integrated measure of soil moisture with a horizontal radius of 15–250 m to depths of tens of centimeters (http://cosmos.hwr.arizona.edu/Probes/StationDat/031/index.php)^58^;
4. Howland has been the location of US EPA (Environmental Protection Agency) CASTNET (Clean Air Status and Trends) monitoring sites for over 25 years (site How132, 1992–2012; site How191, 2011-ongoing), providing long-term measurements of N and S deposition as well as O_3_ concentrations (https://www.epa.gov/castnet);
5. Digital cameras installed on the main and north Howland towers have been used to track vegetation phenology of both evergreen and deciduous species^59^, and imagery and data are publicly available in real time through the PhenoCam Network (http://phenocam.sr.unh.edu).

### ISA-Tab metadata file

Download metadata file.

## Data Availability

The EddyPro software used to generate this dataset is publicly and freely available from the developers (https://www.licor.com/env/support/EddyPro/home.html).

## Online-only Table

**Online-only Table 1:** Headings and description of data columns in the unfiltered EddyPro output file for the tower flux data set derived using gas concentration measurements from the Picarro CRDS gas analyser (US-Ho1_HH_201206060000_201806302330_EP.csv).

Label (units or format)	Description
TIMESTAMP_START (YYYYMMDDHHMM)	*AmeriFlux-format time stamp at beginning of 30-minute averaging period*
TIMESTAMP_END (YYYYMMDDHHMM)	*AmeriFlux-format time stamp at end of 30-minute averaging period*
Year	Calendar year of the measured data
Filename	Name of the raw file (or the first of a set) from which the dataset for the current averaging interval was extracted
date (YYYY-MM-DD)	Date of the end of the averaging period
time (HH:MM)	Time of the end of the averaging period
*DOY*	*Day of year (decimal portion indicates 24* *h fraction)*
*daytime*	*Binary flag where 0* = *nighttime*, *1* = *daytime*, *based on threshold of incident solar radiation (PPFD* ≤ *5* μ*mol m*^−2^ *s*^−1^*)*
file_records	Number of valid records found in the raw file (or set of raw files)
used_records	Number of valid records used for current the averaging period
Tau (kg m^−1^ s^−2^)	Corrected momentum flux
qc_Tau	Quality flag for momentum flux
rand_err_Tau (kg m^−1^ s^−2^)	Random error for momentum flux
H (W m^−2^)	Corrected sensible heat flux
qc_H	Quality flag for sensible heat flux
rand_err_H (W m^−2^)	Random error for momentum flux
LE (W m^−2^)	Corrected latent heat flux
qc_LE	Quality flag latent heat flux
rand_err_LE (W m^−2^)	Random error for latent heat flux
gas_flux (µmol m^−2^ s^−1^)	Corrected gas (CO_2_, H_2_O or CH_4_) flux
qc_gas_flux	Quality flag for (CO_2_, H_2_O or CH_4_) flux
rand_err_gas_flux (µmol s^−1^ m^−2^)	Random error for (CO_2_, H_2_O or CH_4_) flux, if selected
H_strg (W m^−2^)	Estimate of storage sensible heat flux
LE_strg (W m^−2^)	Estimate of storage latent heat flux
gas_strg (µmol s^−1^ m^−2^)	Estimate of storage gas (CO_2_, H_2_O or CH_4_) flux
gas_v-adv (µmol s^−1^ m^−2^)	Estimate of vertical advection gas (CO_2_, H_2_O or CH_4_) flux
gas_molar_density (mmol m^−3^)	Measured or estimated molar density of gas (CO_2_, H_2_O or CH_4_)
gas_mole_fraction (µmol mol^−1^)	Measured or estimated mole fraction of gas (CO_2_, H_2_O or CH_4_)
gas_mixing_ratio (µmol mol^−1^)	Measured or estimated mixing ratio of gas (CO_2_, H_2_O or CH_4_)
gas_time_lag (s)	Time lag used to synchronize gas (CO_2_, H_2_O or CH_4_) time series
gas_def_timelag (T/F)	Flag: whether the reported time lag is the default (T = 1) or calculated (F = 0)
sonic_temperature (K)	Mean temperature of ambient air as measured by the anemometer
air_temperature (K)	Mean temperature of ambient air, as *measured by platinum resistance thermometer*
air_pressure (Pa)	Mean pressure of ambient air, *as measured by the gas analyzer*
air_density (kg m^−3^)	Density of ambient air
air_heat_capactiy (J K^−1^ kg^−1^)	Specific heat at constant pressure of ambient air
air_molar_volume (m^−3^ mol^−1^)	Molar volume of ambient air
ET (mm hour^−1^)	Evapotranspiration flux
water_vapor_density (kg m^−3^)	Ambient mass density of water vapor
e (Pa)	Ambient water vapor partial pressure
es (Pa)	Ambient water vapor partial pressure at saturation
specific_humidity (kg kg^−1^)	Ambient specific humidity on a mass basis
RH (%)	Ambient relative humidity
VPD (Pa)	Ambient water vapor pressure deficit
Tdew (K)	Ambient dew point temperature
u_unrot (m s^−1^)	Wind component along the u anemometer axis
v_unrot (m s^−1^)	Wind component along the v anemometer axis
w_unrot (m s^−1^)	Wind component along the w anemometer axis
u_rot (m s^−1^)	Rotated u wind component (mean wind speed)
v_rot (m s^−1^)	Rotated v wind component (should be zero)
w_rot (m s^−1^)	Rotated w wind component (should be zero)
wind_speed (m s^−1^)	Mean wind speed
max_wind_speed (m s^−1^)	Maximum instantaneous wind speed
wind_dir (degrees)	Direction from which the wind blows, with respect to Geographic or Magnetic north
yaw (degrees)	First rotation angle
pitch (degrees)	Second rotation angle
u* (m s^−1^)	Friction velocity
TKE (m^2^ s^−2^)	Turbulent kinetic energy
L (M)	Monin-Obukhov length
(z-d)/L	Monin-Obukhov stability parameter
bowen_ratio	Sensible heat flux to latent heat flux ratio
T* (K)	Scaling temperature
(footprint) model	Model for footprint estimation *(Kljun et al*. *method*^63^ *generally used [model* = *0]*, *but not suitable for certain atmospheric conditions*, *when Kormann and Meixner method*^64^ *is used instead [model* = *1])*
x_offset (m)	Along-wind distance providing <1% contribution to turbulent fluxes
x_peak (m)	Along-wind distance providing the highest (peak) contribution to turbulent fluxes
x_10% (m)	Along-wind distance providing 10% (cumulative) contribution to turbulent fluxes
x_30% (m)	Along-wind distance providing 30% (cumulative) contribution to turbulent fluxes
x_50% (m)	Along-wind distance providing 50% (cumulative) contribution to turbulent fluxes
x_70% (m)	Along-wind distance providing 70% (cumulative) contribution to turbulent fluxes
x_90% (m)	Along-wind distance providing 90% (cumulative) contribution to turbulent fluxes
un_Tau (kg m^−1^ s^−2^)	Uncorrected momentum flux
Tau_scf	Spectral correction factor for momentum flux
un_H (W m^−2^)	Uncorrected sensible heat flux
H_scf	Spectral correction factor for sensible heat flux
un_LE (W m^−2^)	Uncorrected latent heat flux
LE_scf	Spectral correction factor for latent heat flux
un_gas_flux (µmol s^−1^ m^−2^)	Uncorrected gas (CO_2_, H_2_O or CH_4_) flux
gas_scf	Spectral correction factor for gas (CO_2_, H_2_O or CH_4_) flux
spikes_hf (u/v/w/ts/co2/h2o/ch4/n2)	Hard flags for individual variables for spike test
amplitude_resolution_hf (“”)	Hard flags for individual variables for amplitude resolution
drop_out_hf (“”)	Hard flags for individual variables for drop-out test
absolute_limits_hf (“”)	Hard flags for individual variables for absolute limits
skewness_kurtosis_hf (“”)	Hard flags for individual variables for skewness and kurtosis
skewness_kurtosis_sf (“”)	Soft flags for individual variables for skewness and kurtosis test
discontinuities_hf (“”)	Hard flags for individual variables for discontinuities test
discontinuities_sf (“”)	Soft flags for individual variables for discontinuities test
timelag_hf	Hard flags for gas concentration for time lag test
timelag_sf	Soft flags for gas concentration for time lag test
attack_angle_hf	Hard flag for attack angle test
non_steady_wind_hf	Hard flag for non-steady horizontal test
var_spikes (u/v/w/ts/co2/h2o/ch4)	Number of spikes detected and eliminated for variable var
var_var (u/v/w/ts/co2/h2o/ch4)	Variance of variable var
w/var_cov (ts/co2/h2o/ch4)	Covariance between w and variable var
var_mean (co2/h2o)	Mean value of variable var
TA_1_1_1 (°C)	*Mean temperature of ambient air*, *as measured by platinum resistance thermometer*
P_RAIN_1_1_1 (mm)	*Precipitation falling as rain (may under-estimate precipitation falling as snow)*
NETRAD_1_1_1 (W m^−2^)	*Net radiation (Kipp & Zonen CNR4-4)*
PPFD_IN_1_1_1 (µmol m^−2^ s^−1^)	*Incident photosynthetic photon flux density (Li-Cor LI-190SA)*
TS_1_1_1 (°C)	*Soil temperature at 5 cm depth (Thermocouple)*
WTD_1_1_1 (cm)	*Water table depth (Global Water WL400)*

Reproduced from EddyPro documentation^35^ (following the standard EddyPro naming convention, units, and description) except for text in italics, which indicates expanded description added by the authors of this Data Descriptor. The final six columns report basic environmental and meteorological data, repeated from the filtered AmeriFlux-format dataset.
